# Nanosized Eu^3+^-Doped NaY_9_Si_6_O_26_ Oxyapatite Phosphor: A Comprehensive Insight into Its Hydrothermal Synthesis and Structural, Morphological, Electronic, and Optical Properties

**DOI:** 10.3390/nano14201639

**Published:** 2024-10-12

**Authors:** Madalina Ivanovici, Aleksandar Ćirić, Jovana Periša, Milena Marinović Cincović, Mikhail G. Brik, Abdullah N. Alodhayb, Željka Antić, Miroslav D. Dramićanin

**Affiliations:** 1 National Institute of Research and Development for Electrochemistry and Condensed Matter-INCEMC, 300569 Timisoara, Romania; ivanovicigabriela11@yahoo.com (M.I.); mikhail.brik@ut.ee (M.G.B.); 2 Centre of Excellence for Photoconversion, Vinča Institute of Nuclear Sciences—National Institute of the Republic of Serbia, University of Belgrade, 11000 Belgrade, Serbia; aleksandarciric83@gmail.com (A.Ć.); jburojevic@yahoo.com (J.P.); 3 Vinča Institute of Nuclear Sciences—National Institute of the Republic of Serbia, University of Belgrade, 11000 Belgrade, Serbia; milena@vinca.rs; 4 Department of Physics and Astronomy, College of Science, King Saud University, Riyadh 11451, Saudi Arabia; aalodhayb@ksu.edu.sa

**Keywords:** NaY_9_Si_6_O_26_, oxyapatite, europium ions, nanophosphors, amorphous-to-crystalline transformation

## Abstract

Detailed analysis covered the optical and structural properties of Eu^3+^-doped NaY_9_Si_6_O_26_ oxyapatite phosphors, which were obtained via hydrothermal synthesis. X-ray diffraction patterns of NaY_9_Si_6_O_26_:xEu^3+^ (x = 0, 1, 5, 7, 10 mol% Eu^3+^) samples proved a single-phase hexagonal structure (*P63/m* (176) space group). Differential thermal analysis showed an exothermic peak at 995 °C attributed to the amorphous to crystalline transformation of NaY_9_Si_6_O_26_. Electron microscopy showed agglomerates composed of round-shaped nanoparticles ~53 nm in size. Room temperature photoluminescent emission spectra consisted of emission bands in the visible spectral region corresponding to ^5^D_0_ → ^7^F*_J_* (*J* = 0, 1, 2, 3, 4) *f-f* transitions of Eu^3+^. Lifetime measurements showed that the Eu^3+^ concentration had no substantial effect on the rather long ^5^D_0_-level lifetime. The Eu^3+^ energy levels in the structure were determined using room-temperature excitation/emission spectra. Using the ^7^F_1_ manifold, the Nv-crystal field strength parameter was calculated to be 1442.65 cm^−1^. Structural, electronic, and optical properties were calculated to determine the band gap value, density of states, and index of refraction. The calculated direct band gap value was 4.665 eV (local density approximation) and 3.765 eV (general gradient approximation). Finally, the complete Judd–Ofelt analysis performed on all samples confirmed the experimental findings.

## 1. Introduction

Compounds with the general formula Me_10_(TO_4_)_6_O_2_ belong to apatite-type materials with a specific arrangement of metal cations, tetrahedral units, and oxygen atoms. In these materials, Me stands for metal cations, usually alkali and alkaline metals and/or rare earth elements; (TO_4_)_6_ refers to six tetrahedral units where T is a tetrahedral metal (like silicon or germanium) each surrounded by four oxygen atoms; and O_2_ represents two additional oxygen atoms that are not part of the tetrahedral units. Depending on the metal cations and the tetrahedral element T, this group of materials can find applications in: (i) materials science where such compounds might be of interest for their structural properties, which can be useful in high-temperature ceramics or advanced materials; (ii) catalysis where they may be used as catalysts or catalyst supports due to their stable and unique crystal structures; and/or (iii) phosphors for lighting in LEDs, OLEDs, and other display technologies if the material is luminescent. Their multiple combinations lead to the design of complex materials that are studied for important applications such as high oxide ion conductivity and photoluminescence, immobilizing radioactive waste, laser research, solid-state lighting, oxide fuel cell development, biomaterials, etc. [1,2,3,4,5,6].

Alkaline metal/rare earth orthosilicates are a family of apatite oxide materials called oxyapatites that have gained interest, especially for their luminescent properties. Among them, sodium yttrium orthosilicate is an oxyapatite of the NaY_9_Si_6_O_26_ general formula featuring a structure where yttrium is incorporated into the apatite-like framework, and sodium ions are included to balance the charge. The presence of trivalent yttrium ions in the structure makes this host suitable for activation with other rare earths and optically active luminescent materials design. Only a few studies have published the synthesis and characterization of NaY_9_Si_6_O_26_ doped with activating ions. Chuai et al. investigated the luminescence of Eu^3+^-, Tb^3+^-, Dy^3+^-, and Pb^2+^-doped NaY_9_Si_6_O_26_ obtained through the sol–gel technique [7]. The authors concluded that lanthanides occupy both sites in the oxyapatite structure and that white light emission of Dy^3+^-doped samples improve when combined with Pb^2+^ and Gd^3+^ co-dopants. Kang et al. reported the conventional solid-state synthesis of NaY_9_Si_6_O_26_ doped with Yb^3+^, an NIR-emitting material serving as a potential state of the art candidate for advanced anti-counterfeiting utilization [8]. Zheng et al. reported color-tunable emission of Eu^3+^-, Ce^3+^-, and Eu^3+^/Ce^3+^-doped NaY_9_Si_6_O_26_ obtained with a conventional solid-state reaction [9]. The authors reported emission in the blue wavelength range for the Ce^3+^-doped sample, and in the reddish-orange wavelength for the Eu^3+^-doped sample while the emission of the Eu^3+^/Ce^3+^ sample changed from blue to orange-reddish by changing the Eu^3+^ concentration. Zheng et al. also reported color tuning in Bi^3+^- and Eu^3+^-doped/co-doped NaY_9_Si_6_O_26_ obtained via a solid-state reaction as a solution to obtain phosphors with white light emission. The proper concentration and ratio of Bi^3+^/Eu^3+^ activating ions resulted in white light emission, while different concentrations of solely added Bi^3+^ and Eu^3+^ could alter the emission from blue to an orange-red color [10]. In addition, Brgoch et al. reported rapid microwave preparation of blue-emitting cerium-substituted NaY_9_Si_6_O_26_ using active carbon as a microwave susceptor [11].

The correlation between structure and optical properties is essential when defining the host material and related efficiency for phosphor applications. Therefore, this research aims to investigate and present detailed structural and optical properties of Eu^3+^-doped NaY_9_Si_6_O_26_ oxyapatite phosphor obtained by hydrothermal synthesis. The hydrothermal technique offers several advantages over other synthesis methods, such as: (1) a controlled environment—the high temperature and pressure in hydrothermal conditions allow for better control over the reaction environment, leading to more uniform products; (2) higher solubility—unlike the solid-state method that struggles with complex materials or a precipitation method limited to materials that can be precipitated from solution, materials that are difficult to dissolve at room temperature can be effectively synthesized in a hydrothermal setup, enabling the formation of complex compounds; (3) good crystallinity—due to slow growth rates hydrothermal synthesis often results in high crystallinity of the final product compared to room/mild temperature techniques like precipitation; (4) versatility—this method can be used to synthesize a wide range of complex materials, making it more versatile when compared to solid-state or precipitation; (5) eco-friendly—hydrothermal processes often use water as a solvent, reducing the need for harmful organic surfactants usually needed in precipitation; (6) scalability—unlike solid-state and precipitation methods where quality can vary significantly with the batch, hydrothermal synthesis can be scaled up easily for industrial applications, allowing for the production of larger quantities of good quality materials; and (7) low energy consumption—compared to other methods, especially solid-state reactions, hydrothermal synthesis is more energy-efficient.

Thermal analysis was performed to determine the proper calcination temperature for oxyapatite phase transformation. Structural and photoluminescent measurements were analyzed and correlated. Structural, electronic, and optical properties were calculated to determine band gap value, density of states, and index of refraction. Finally, Judd–Ofelt analysis calculated the intensity parameters for electric dipole transitions, allowing an estimate of their radiative properties. To the extent of our knowledge, there are no data on the temperature needed for the amorphous-to-crystalline transformation of the NaY_9_Si_6_O_26_ oxyapatite host nor an index of refraction values, both being very important for the potential application of alkaline metal/rare earth orthosilicates.

## 2. Materials and Methods

### 2.1. Hydrothermal Synthesis of Undoped and Eu^3+^-Doped Apatite Samples

A total of five samples of undoped and Eu^3+^-doped apatite—NaY_9_Si_6_O_26_:xEu^3+^ (x = 0, 1, 5, 7, 10 mol% Eu^3+^; molecular formula of each sample is given in Table 1) were synthesized via the hydrothermal method [12,13]. The proper amounts of Na_2_SiO_3_ × 9H_2_O (Sigma Aldrich, 98%, St. Louis, MO, USA), Y(NO_3_)_3_ × 6H_2_O (Sigma Aldrich, 99.8%), and Eu(NO_3_)_3_ × 6H_2_O (Alfa Aesar, 99.9%, Haverhill, MA, USA) were dissolved in deionized water leading to the formation of the aqueous precursor solutions. That way, concentrations of 0.299 mol/dm^3^ for the Y and Eu precursors and 0.225 mol/dm^3^ for the Si precursor solution were prepared. The Si solution was added in drops under intense magnetic stirring to the Y/Eu solution (molar ratio Eu/Y:Si = 1.33:1), and the pH of the precursor mixture was tuned to 13 by the dropwise addition of sodium hydroxide solution (6 mol/dm^3^). The final mixture solution was stirred for another 3 h and then transferred to the PTFE liner of the hydrothermal reactor using a filling ratio of 30%. The hydrothermal reaction was carried out in the hydrothermal autoclave reactor at 230 °C for 12 h; the precursor was then separated by centrifugation, washed with water five times, and dried at 80 °C for 2 h, resulting in an amorphous material. The crystalline samples were obtained by calcinating the amorphous materials for 12 h at 1100 °C.

### 2.2. Characterization of the Apatite Materials

Thermal stability was examined by non-isothermal thermo-gravimetric analysis (TG) and differential thermal analysis (DTA), simultaneously, using a SETARAM SETSYS Evolution 1750 instrument (heating rate −20 °C/min in a dynamic air atmosphere; flow rate −40 cm^3^/min; temperature range 30–1300 °C). The mass of the sample was about 5 mg. The crystal structures of the obtained phosphors were looked at carefully with an X-ray diffractometer (XRD) from Rigaku SmartLab (Cu-K_α1,2_ radiation, λ = 0.1540 nm) at room temperature. The experimental conditions for measurements were as follows: 2θ range of 20°–80°, with a step size of 0.02°, and a counting time of 10°/min. Conclusions on the structural study (unit cell parameters, crystal coherence size, micro strain values, and data fit parameters) were attained using the built-in PDXL2 software (v2.1). The average particle size was calculated using ImageJ software (https://imagej.net). The morphology was examined by a TESCAN MIRA3 field emission scanning electron microscope (FE-SEM) (TESCAN, Brno, Czechia), with the samples coated using a thin layer of Au, and by transmission electron microscope (TEM) JEOL JEM1011 operated at an accelerating voltage of 100 kV (JEOL, Frenchs Forest, NSW, Australia). The sample’s UV–VIS diffuse reflection spectrum was recorded with a Shimadzu UV-3600 UV-VIS-NIR spectrophotometer with BaSO_4_ used as the reflectance standard (Shimadzu, Kyoto, Japan). Photoluminescence measurements were carried out using a Fluorolog-3 Model FL3-221 spectrofluorometer system (Horiba Jobin-Yvon, Longjumeau, France) equipped with an R928 PMT detector. Emission and excitation spectra were corrected for the lamp spectral intensity and detector sensitivity. Excited-state lifetime measurements were carried out using the Rohde&Schwarz RTC1002 two-channel oscilloscope paired with the Hamamatsu H10722-20 photomultiplier tube, and by exciting the samples with a square wave modulated Ocean Insight fiber-coupled LED (LSM-405A LED Light Source, Winter Park, FL, USA) controlled by an Ocean Insight LDC-1 Single Channel LED Controller.

## 3. Results and Discussion

### 3.1. Thermal Stability (TGA) and Differential Thermal Analysis (DTA)

**Figure 1:**
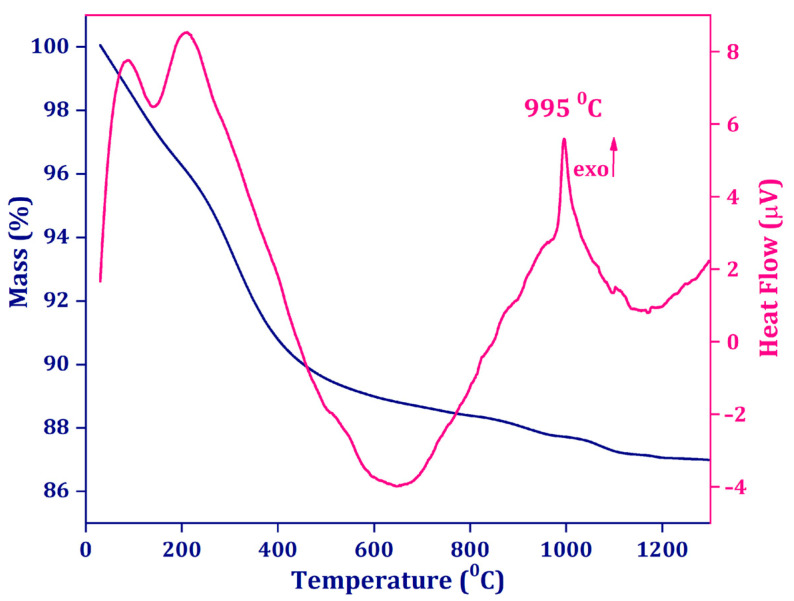
Thermogravimetry (TG, blue) and differential thermal analysis (DTA, pink) curves for undoped NaY_9_Si_6_O_26_ precursor prepared via the hydrothermal method.

The hydrothermal method used to obtain the desired materials resulted in amorphous apatite samples which, under appropriate sintering, transformed to crystalline NaY_9_Si_6_O_26_. The undoped amorphous precursor was investigated to establish the adequate calcination temperature needed for transformation to the apatite phase. DTA analysis showed an exothermic peak at 995 °C that could be attributed to the transformation of amorphous NaY_9_Si_6_O_26_ to the crystalline phase NaY_9_Si_6_O_26_. At the same time, the TG curve showed ~13% total mass loss that could be attributed to the vaporization of adsorbed water and residual molecules. At the same time, the initial TG curve showed a sharp mass loss that could be attributed to the fast vaporization of adsorbed water and residual molecules. The total mass loss was found to be ~13%. According to the TG/DTA analysis results, calcination at 1100 °C was chosen to complete the amorphous-to-crystalline transformation (Figure 1).

Table 2 presents a comparison of the obtained amorphous-to-crystalline transformation temperature with the values found in the literature for some rare earth silicates.

### 3.2. Structural Characterization

**Figure 2:**
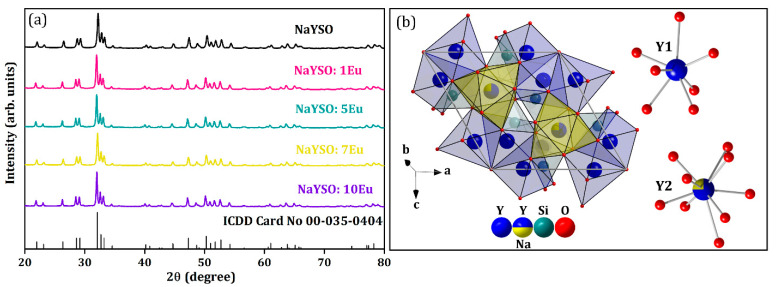
(**a**) XRD patterns of NaY_9−x_Eu_x_Si_6_O_26_ materials with standard ICDD Card No. 00-035-0404; (**b**) representation of NaY_9_Si_6_O_26_ crystal structure and coordination environment of Y1 and Y2 atoms in the NaY_9_Si_6_O_26_ crystal lattice designed with DIAMOND Release 4.6.8. Crystal Impact GbR software (version 4.0).

Figure 2a shows the X-ray diffraction pattern of NaY_9_Si_6_O_26_:xEu^3+^ (x = 0, 1, 5, 7, 10 mol% Eu^3+^) samples depicted with a corresponding ICDD Card No. 00-035-0404. X-ray diffraction analysis verified a single-phase hexagonal structure (space group: *P63/m* (176)) with no other phase peaks or traces of impurities signifying that dopant Eu^3+^ ions have been efficiently incorporated into the NaY_9_Si_6_O_26_ host lattice owing to similar sizes between Y^3+^ and Eu^3+^ ions [21]. PDXL2 software was used to calculate the average crystallite size and structural parameters presented in Table 3. The initial parameters for the evaluation were extracted from Reference [22]. The calculated mean crystallite size for all the samples was in the nanometer region (~14–25 nm).

As seen from Table 3 the value of lattice constant c becomes larger with the increase of Eu^3+^ concentration. When Y^3+^ is replaced with Eu^3+^ within the crystal structure, the significant increase in the lattice parameter c rather than a = b can be explained by several factors such as: (a) ionic radius—Eu^3+^ has a larger ionic radius than Y^3+^ which can lead to an expansion of the unit cell in the vertical direction (along the c-axis); (b) crystal field effects—the different electronic configurations and interactions of Eu^3+^ compared to Y^3+^ can alter the crystal field, which affects the bond lengths and angles, particularly in the axial direction, which might preferentially expand the c-parameter; (c) phase stability—the introduction of Eu^3+^ may stabilize certain orientations within the crystal structure that are more favorable in the vertical direction, leading to an increase in c without a corresponding increase in a = b. So, the significant increase in the c lattice parameter upon replacing Y^3+^ with Eu^3+^ in a hexagonal crystal structure is primarily driven by the larger ionic radius of Eu^3+^, changes in crystal field interactions, and potentially different bonding characteristics that favor expansion along the c-axis.

There are two distinct crystallographic positions for the Y^3+^ in the NaY_9_Si_6_O_26_ crystal structure (Figure 2b): one is coordinated by seven oxygen atoms (Y1), and the other one by nine (Y2), while the silicon atom is positioned in a tetrahedral site. In the nine-coordinated site, one quarter of the Y^3+^ position is occupied by Na^+^ atoms, thus preserving the charge balance [23].

### 3.3. Microstructural Characterization by Electron Microscopy (SEM and TEM)

**Figure 3:**
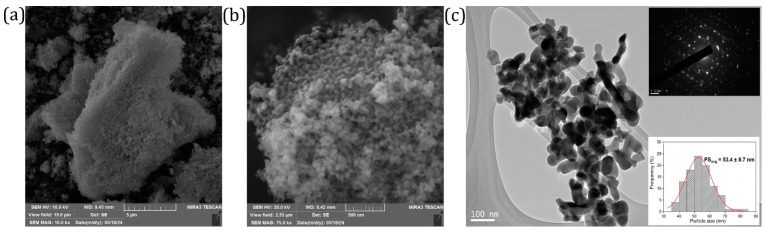
SEM images of the representative NaYSO:1Eu powder sample at different magnifications: (**a**) ×10,000, (**b**) ×75,000, (**c**) TEM image of the representative NaYSO:1Eu powder sample with electron diffraction pattern and particle size distribution histogram as insets.

It is generally acknowledged that in nanoparticles with a large specific surface area, luminescence characteristics are affected by the particles’ size, which ultimately influences excitation efficiency and emission. The SEM images captured at low magnification illustrate that the representative NaYSO:1Eu powder sample tends to form agglomerates (Figure 3a). At higher magnification, the surface morphology shows that the particles can be classed as round-shaped nano-sized particles (Figure 3b). Figure 3c illustrates the microstructure at a local scale, examined through transmission electron microscopy, and shows the presence of sphere-like nanoparticles. The TEM images used for the average particle size analysis considered over 70 particles, using the largest axis of the grain. From the inset in Figure 3c, it can be concluded that the most frequent nanoparticle size is in the 45 to 60 nm range with an average particle size of ~53 nm in diameter. The upper inset in Figure 3c shows a ring electron diffraction pattern as evidence of the polycrystalline nature of the material. At the same time, the rings’ grainy form is linked to the finding that the constituent crystallites have a size of ~20 nm. The ring pattern is formed due to diffraction from polycrystalline material because diffracting planes are oriented randomly in all possible directions. This agrees with the crystallite size obtained from XRD measurements (~20 nm) and particle size obtained from TEM (45–60 nm range) showing that each particle is composed of approximately 2–3 crystallites.

### 3.4. Calculations of the Structural and Electronic Properties

Calculations of the structural and electronic properties of NaY_9_Si_6_O_26_ were performed using the CASTEP module of Materials Studio [24]. The general gradient approximation (GGA) [25] and local density approximation (LDA) [26] were used to treat the exchange-correlation effects. The plane wave basis set cut-off was 370 eV; the k-points grid was 3 × 3 × 4. The electronic configurations for the atoms in the unit cell were 2s^2^2p^6^3s^1^ for Na, 3s^2^3p^2^ for Si, 4s^2^4p^6^4d^1^5s^2^ for Y, and 2s^2^2p^4^ for O.

**Figure 4:**
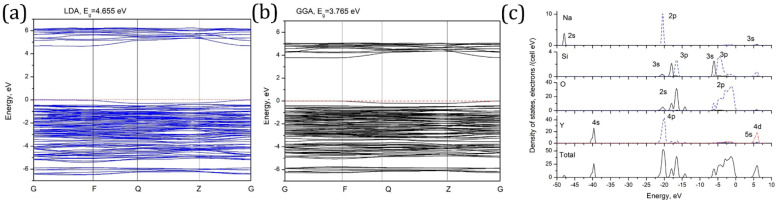
(**a**,**b**) Calculated band structure of NaY_9_Si_6_O_26_. The coordinates of the special points of Brillouin zone are (in units of the reciprocal lattice unit vectors): G (0, 0, 0); F (0, ½, 0); Q (0, ½, ½); Z (0, 0, ½), and (**c**) calculated density of states diagrams for NaY_9_Si_6_O_26_.

Figure 4a,b show the calculated (in both approximations) band structure of the studied material. The calculated direct band gap values are 4.665 eV (LDA) and 3.765 eV (GGA).

Very low dispersion of the calculated electronic states, which follows from nearly flat electronic bands, indicates low mobility of the charge carriers. The origin of the calculated bands can be clarified with the help of the density of states diagrams (Figure 4c). The conduction band consists mainly of the Y 4d and Na 3s states. The upper part of the valence band is made by the oxygen 2p states, and highly hybridized 3s-3p states of silicon in four-fold coordination (sp^3^ hybridized states) are mainly at the bottom of the valence band. Several narrow bands between −22 eV and −13 eV are made by the oxygen 2s and the same highly hybridized Si 3s-3p states. Finally, at about −48 eV and −40 eV very deep Na 2s and Y 4s states, respectively, can be identified.

### 3.5. Optical Spectroscopy

**Figure 5:**
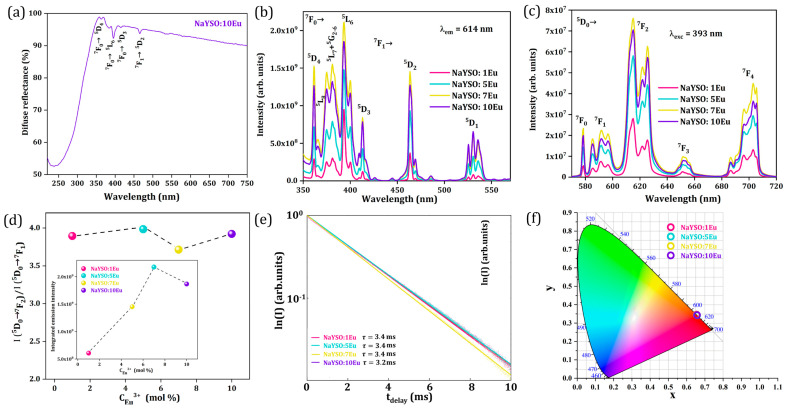
(**a**) Diffuse reflectance spectrum of NaYSO:10Eu sample. Ambient temperature photoluminescence of NaY_9_Si_6_O_26_:xEu^3+^ (x = 1, 5, 7, 10 mol% Eu^3+^) samples: (**b**) excitation spectra under λ_em_ = 614 nm; (**c**) emission spectra under λ_exc_ = 393 nm; (**d**) asymmetry ratio as a function of Eu ions concentration with integrated emission intensity as a function of Eu^3+^ ions concentration given as Inset; (**e**) lifetime decay curves as a function of Eu^3+^ ions concentration; (**f**) CIE diagram with calculated coordinates.

The diffuse reflectance spectrum of the NaYSO:10Eu sample given in Figure 5a is recorded in the 220–700 nm range. A broad absorption below 250 nm is attributed to the charge transfer band while peaks at longer wavelengths are attributed to the *f-f* transition of Eu^3+^ ions.

The photoluminescence excitation spectra of all NaY_9_Si_6_O_26_:xEu^3+^ (x = 1, 5, 7, 10 mol% Eu^3+^) samples, recorded in the 350–575 nm (λ_em_ = 614 nm) range, are given in Figure 5b, showing emission lines of typical transitions within the 4*f*^6^ configuration of Eu^3+^. Excitation into the ^5^L_6_ (393 nm) energy level of Eu^3+^ is picked for observing the emission spectra since it is the most intense peak in the excitation spectra. Upon excitation, distinct emission bands in the visible spectral region corresponding to ^5^D_0_ → ^7^F_J_ (J = 0, 1, 2, 3, and 4) *f-f* transitions, placed around 578 nm, 592 nm, 615 nm, 652 nm, and 703 nm, respectively, are visible (see Figure 5c).

As mentioned above, there are two different Y^3+^ sites with unequal coordination numbers in the silicate apatite structure [7], one having nine-coordination with *C_3_* symmetry and the other having seven-coordination with *Cs* symmetry, both being non-centrosymmetric. Such low symmetry stimulates the splitting of energy levels into a large number of Stark sublevels [27]. According to the Laport rule, the Eu^3+^ ion, with an [Xe]4f^6^ electronic configuration, has only magnetic-dipole transitions in a centrosymmetric site, whereas if in a non-centrosymmetric site, both magnetic-dipole and induced electric-dipole transitions are probable.

The ^5^*D*_0_ → ^7^*F*_1_ is a magnetic-dipole transition that does not depend on the local environment. However, the ^5^*D*_0_ → ^7^*F*_2_ electric-dipole transition is hypersensitive and highly dependent on changes in the local environment around the Eu^3+^ ions. Theoretically, when the Eu^3+^ ions occupy non-centrosymmetric sites, the emission spectrum shows a more intense ^5^*D*_0_ → ^7^F_2_ transition than the ^5^*D*_0_ → ^7^*F*_1_, which agrees with our experimental observations. In addition, the ratio of the integrated intensity of the ^5^*D*_0_ → ^7^*F*_2_ and ^5^*D*_0_ → ^7^*F*_1_ transitions, known as the asymmetry ratio, can be considered indicative of the reduction of symmetry of the coordination environment around the Eu^3+^ ion and is given by Equation (1):

(1) R=ID 50→F 72ID 50→F 71

The asymmetry ratio values obtained from the emission spectra are presented in Figure 5d showing no significant change with Eu^3+^ concentration. The high value of the asymmetry ratio as a function of dopant ion concentration indicates low symmetry of the Eu^3+^ surroundings in the NaY_9_Si_6_O_26_ apatite host. Also, no significant change with Eu^3+^ concentration reveals that the degree of distortion of the local symmetry around Eu^3+^ is similar in all the samples in the series produced, as indicated by XRD analysis. The inset in Figure 5d presents the integrated emission intensity as a function of Eu^3+^ concentration showing that 7 mol% Eu^3+^ is the optimal doping concentration in the NaY_9_Si_6_O_26_ host lattice.

The photoluminescent lifetime decay curves of all Eu^3+^-doped NaYSO samples recorded at ambient temperature can be seen in Figure 5e. The values of the lifetime (*τ*) were obtained after the data were fitted to a simple single exponential function. Even though two distinct crystallographic positions for Y^3+^ can host Eu^3+^ ions, our experimental data reveal single exponential decay curves. This can be explained either by both Y sites being non-centrosymmetric and having similar C_3_ and C_s_ symmetry or, most likely, by the similar lifetime values for each of the two Y sites that are not distinguishable by our integrated intensity decay measurement. All samples have similar, rather long lifetime values calculated to be in the 3.2 to 3.4 ms range.

Chromaticity coordinates (x, y) on the CIE chromaticity diagram, which represents a two-dimensional color space encompassing all colors visible to the human eye, can be utilized to quantify apparent color. We derived the CIE chromaticity coordinates from the photoluminescent spectra to evaluate the color of the synthesized samples, as shown in Figure 5f. For all the samples, CIE coordinates are almost identical (x = 0.65, y = 0.34; λ_dom_ = 614 nm; color purity = 93.6%) and placed in the orange-red portion of the diagram, confirming that there is no significant change in the local symmetry around Eu^3+^ across the series and consequently in the emission spectra.

The energy levels of trivalent Eu^3+^ ions in the structure were determined using room temperature excitation and emission spectra and are given in Table 4. Based on the whole luminescence spectra and the ratio of the ^5^D_0_ → ^7^F*_J_* (*J* = 0–4) transitions, it is assumed that the Eu^3+^ ion occupies both sites. Also, using the ^7^F_1_ manifold, we carried out a simplified phenomenological crystal field calculation by considering only the second-order crystal field B20 (51.46 cm^−1^), and B22 (−642.6 cm^−1^) parameters to calculate the crystal field strength parameter Nv (1442.65 cm^−1^) [28], using the simplified Equation (2) given by Monteil and Zhu [29,30].

(2) $$N_{v}(B_{2q})=\sqrt{4\pi (B_{20}^{2}+{2B}_{22}^{2})/5}$$

### 3.6. The Judd–Ofelt Calculation

**Figure 6:**
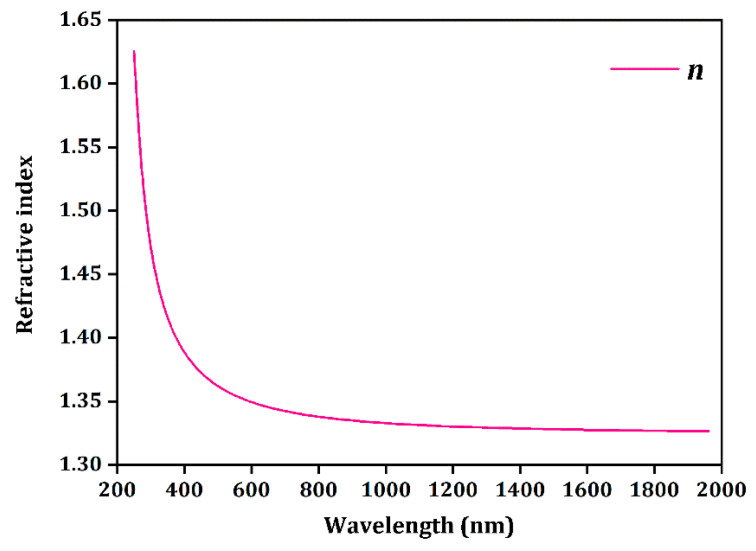
Calculated refractive index of NaY_9_Si_6_O_26_.

To perform Judd–Ofelt calculations, the refractive index of NaY_9_Si_6_O_26_ is determined using the structural and electronic property calculations given above. Figure 6 presents the calculated index of refraction values as a function of wavelength.

The Judd–Ofelt theory is a framework for understanding the optical behavior of rare earth ions in different host materials. It allows for the calculation of intensity parameters for electric dipole transitions, aiding in the prediction of their absorption and emission spectra. In the case of the Eu^3+^ ion, these parameters can be derived from the emission spectrum of Eu^3+^ using the formula [31]:

(3) $$\Omega_{\lambda }=\frac{D_{1}}{e^{2}U^{\lambda }}{\left(\frac{{\bar{\lambda }}_{\lambda }}{{\bar{\lambda }}_{1}}\right)}^{3}\frac{9n_{1}^{3}}{n_{\lambda }{\left(n_{\lambda }^{2}+2\right)}^{2}}\frac{I_{\lambda }}{I_{1}},\;\lambda =2, 4$$

Here, *e* denotes the elementary charge, *U^λ^* represents the squared reduced matrix elements, *n* is the refractive index at the wavelength of the transition, and *I* is the integrated intensity. The transition ^5^D_0_ → ^7^F_1_ is a purely magnetic dipole transition, meaning its dipole strength D_MD_ remains unaffected by the host matrix. The term $\bar{\lambda }$ corresponds to the average wavelength of the emission. The subscripts λ indicate electric dipole transitions.

The Judd–Ofelt Ω_6_ parameter can be best determined from the excitation spectrum of Eu^3+^ using the formula [32]:

(4) $$\Omega_{6}=\frac{n_{6}n_{1}}{{\left(n_{6}^{2}+2\right)}^{2}}\frac{{\bar{\lambda }}_{6}}{{\bar{\lambda }}_{1}}\frac{D_{1}}{e^{2}U^{6}}\frac{I_{6}}{I_{1}}$$

In this context, index 6 corresponds to excitation from the ground level to ^5^L_6_, and 1 for the pure magnetic dipole transition to ^5^D_1_.

Using the Judd–Ofelt parameters, we can derive key quantities relevant to the practical applications of luminescent materials. The radiative transition probabilities, or rates of spontaneous emission, are given by [33]:

(5) $$A_{\lambda }=\frac{64\pi^{4}}{3h}\frac{10^{7}}{{\bar{\lambda }}_{\lambda }^{3}}\frac{n_{\lambda }{\left(n_{\lambda }^{2}+2\right)}^{2}}{9}e^{2}\Omega_{\lambda }U^{\lambda }$$

(6) $$A_{MD}=\frac{64\pi^{4}}{3h}\frac{10^{7}}{{\bar{\lambda }}_{MD}^{3}}n_{MD}^{3}D_{MD}$$

where *h* stands for the Planck constant. The emission branching ratio is defined as:

(7) $$\beta_{\lambda }=\frac{A_{\lambda }}{A_{1}+A_{2}+A_{4}}$$

Note that *A*_6_ is neglected as its intensity is very low and because the transition ^5^D_0_ → ^7^F_6_ lies outside the visible range. The radiative lifetime is the inverse of the total radiative transition probability:

(8) $$\tau_{R}={\left(A_{1}+A_{2}+A_{4}\right)}^{-1}$$

The intrinsic quantum yield can then be calculated from the ratio of the observed and radiative lifetimes:

(9) $$\eta =\frac{\tau }{\tau_{rad}}$$

A complete Judd–Ofelt analysis was performed on all concentrations and the results are summarized in Table 5. The Ω_2_ parameter, which is related to the degree of symmetry and covalency of the Eu^3+^ ion, is unchanged with the Eu^3+^ concentration, while the Ω_4_ and Ω_6_ parameters related to the long-range effects and rigidity of the matrix [34] see a small decrease with rising concentration. According to the branching ratio and the radiative transition probability, the hypersensitive ^5^D_0_ → ^7^F_2_ transition is dominant in the emission spectrum. The concentration has no significant impact on the rather long radiative lifetime of ^5^D_0_ emissions, as confirmed by experimental data given previously in Figure 5e.

## 4. Conclusions

This study gives a comprehensive insight into Eu^3+^-doped NaY_9_Si_6_O_26_ oxyapatite phosphor—its synthesis, structural, morphological, electronic, and optical properties. It is shown that with hydrothermal synthesis, it is possible to obtain single-phase hexagonal NaY_9_Si_6_O_26_ oxyapatite nanophosphors. Differential thermal analysis shows that, in the NaY_9_Si_6_O_26_ host material, amorphous-to-crystalline transformation is somewhat lower compared to simple orthosilicates (Y_2_SiO_5_ and Y_2_Si_2_O_7_) and comparable to similar, apatite-type silicates (i.e., La_10_Si_6_O_27_). Photoluminescent measurements show typical orange-red emission of trivalent Eu^3+^ showing that 7 mol% Eu^3+^ is the optimal doping concentration in the NaY_9_Si_6_O_26_ host lattice. However, lifetime measurements show that the Eu^3+^ concentration has no significant impact on the rather long radiative lifetime of ^5^D_0_ emission. The findings presented here can be used to further develop alkaline metal/rare earth orthosilicate phosphors that meet specific needs in technology and industry.

## Figures and Tables

**Table 1:** Chemical formulas of the synthesized NaY_9_Si_6_O_26_:xEu^3+^ (x = 0, 1, 5, 7, 10 mol% Eu^3+^) samples.

Sample Code	Eu^3+^ (mol %)	Molecular Formula
NaYSO	0	NaY_9_Si_6_O_26_
NaYSO:1Eu	1	NaY_8.91_Eu_0.09_Si_6_O_26_
NaYSO:5Eu	5	NaY_8.55_Eu_0.45_Si_6_O_26_
NaYSO:7Eu	7	NaY_8.37_Eu_0.63_Si_6_O_26_
NaYSO:10Eu	10	NaY_8.1_Eu_0.9_Si_6_O_26_

**Table 2:** Comparison of the obtained amorphous-to-crystalline transformation temperature with the values found in the literature.

Material	Synthesis	Structure	Total Weight Loss	Crystallization T (°C)	Reference
Y_2_SiO_5_	Sol-gel	Monoclinic	~29.3%	~1040	[14]
Y_2_SiO_5_	Sol-gel	Monoclinic X1—Y_2_SiO_5_ X2—Y_2_SiO_5_	~30%	~1010 ~1360	[15]
Y_2_SiO_5_	Sol-gel	Monoclinic X1—Y_2_SiO_5_ X2—Y_2_SiO_5_	~70%	~1025 ~1350	[16]
Y_2_Si_2_O_7_	Oxalate gel	Monoclinic α—Y_2_Si_2_O_7_	~60%	~1065	[17]
La_10_Si_6_O_27_	Precipitation	Hexagonal	~21.4%	~902	[18]
La_10_Si_6_O_27_	Sol-gel	Hexagonal	~48.4%	~959	[19]
La_9.5_Si_6_O_26.25_	Co-precipitation	Hexagonal	~26.5%	~883	[20]
La_9.33_Si_6_O_26_	Sol-gel	Hexagonal	~48.4%	~874	[19]
**NaY_9_Si_6_O_26_**	**Hydrothermal**	**Hexagonal**	**~13%**	**~995**	**This work**

**Table 3:** Selected structural parameters of the synthesized NaY_9−x_Eu_x_Si_6_O_26_ samples. CS—Crystallite size; Rwp—the weighted profile factor; Rp—the profile factor; Re—the expected weighted profile factor; and GOF—the goodness of fit.

ICDD Card 00-035-0404	NaYSO	NaYSO: 1Eu	NaYSO: 5Eu	NaYSO: 7Eu	NaYSO: 10Eu
**a = b (Å)**	9.3398 (10)	9.3539 (13)	9.3504 (7)	9.3483 (15)	9.3511 (5)
**c (Å)**	6.7508 (8)	6.7571 (10)	6.7610 (5)	6.7611 (11)	6.7769 (5)
**CS (Å)**	181 (2)	212 (3)	233 (3)	219 (2)	282 (3)
**Strain**	0.28 (5)	0.23 (6)	0.21 (4)	0.22 (4)	0.17 (3)
**Rwp**	12.56	12.37	10.69	9.86	9.69
**Rp**	8.86	8.60	7.46	7.0	6.78
**Re**	3.35	3.17	3.13	3.13	2.99
**GOF**	3.7449	3.8971	3.4905	3.2399	3.2399

**Table 4:** Energy levels of Eu^3+^ in the hexagonal NaY_9_Si_6_O_26_.

	^7^F_0_	^7^F_1_	^7^F_2_	^7^F_3_	^7^F_4_	
	0	207	1026	1854	2719	
		380	1209	1937	2804	
[cm^−1^]		522	1299	1984	2928	
				2042	3061	
					3112	
**^5^D_0_**	**^5^D_1_**	**^5^D_2_**	**^5^D_3_**	**^5^L_6_**	**^5^L_7_ + G_2–6_**	**^5^L_8_, ^5^D_4_**
17,286	18,657	21,322	24,213	25,000	26,110	27,322
	18,850	21,575	24,420	25,413	26,247	27,662
	19,029				26,667	

**Table 5:** Judd–Ofelt analysis of NaY_9_Si_6_O_26_ doped with various concentrations of Eu^3+^.

C [%]	Ω_2_∙10^20^ [cm^2^]	Ω_4_∙10^20^ [cm^2^]	Ω_6_∙10^20^ [cm^2^]	A_1_ [s^−1^]	A_2_ [s^−1^]	A_4_ [s^−1^]	β_1_ [%]	β_2_ [%]	β_4_ [%]	τ_R_ [ms]	τ [ms]	η [%]
1	6.5	6.51	1.31	35.5	139.4	68.1	14.6	57.4	28	4.1	3.4	83
5	6.66	6.45	0.69	35.5	142.7	67.4	14.5	58.1	27.4	4.1	3.4	83
7	6.22	6.37	0.632	35.5	133.2	66.6	15.1	56.6	28.3	4.3	3.4	79
10	6.57	6.05	0.494	35.5	140.6	63.2	14.8	58.7	26.4	4.2	3.2	76

## Data Availability

The original contributions presented in the study are included in the article. Further inquiries can be directed to the corresponding authors.

## References

[1] Ptáček P. (2016). Apatites and Their Synthetic Analogues-Synthesis, Structure, Properties and Applications.

[2] Anil, Kumar B., Barwar A., Barbar S.K. (2024). Structural, Optical and Photoluminescence Characteristics of Apatite Type Lanthanide Silicates. J. Mol. Struct..

[3] Kobayashi K., Sakka Y. (2014). Rudimental Research Progress of Rare-Earth Silicate Oxyapatites: Their Identification as a New Compound until Discovery of Their Oxygen Ion Conductivity. J. Ceram. Soc. Jpn..

[4] Neeway J.J., Asmussen R.M., McElroy E.M., Peterson J.A., Riley B.J., Crum J.V. (2019). Kinetics of Oxyapatite [Ca_2_Nd_8_(SiO_4_)_6_O_2_] and Powellite [(Ca,Sr,Ba)MoO_4_] Dissolution in Glass-Ceramic Nuclear Waste Forms in Acidic, Neutral, and Alkaline Conditions. J. Nucl. Mater..

[5] Liu H., Liao L., Pan X., Su K., Shuai P., Yan Z., Guo Q., Mei L. (2022). Recent Research Progress of Luminescent Materials with Apatite Structure: A Review. Open Ceram..

[6] Sumathi S., Gopal B. (2015). A New Insight into Biomedical Applications of an Apatite like Oxyphosphate–BiCa_4_(PO_4_)_3_O. Ceram. Int..

[7] Chuai X.H., Zhang H.J., Li F.S., Lu S.Z., Lin J., Wang S.B., Chi-Chou K. (2002). Synthesis and Luminescence Properties of Oxyapatite NaY_9_Si_6_O_26_ Doped with Eu^3+^, Tb^3+^, Dy^3+^ and Pb^2+^. J. Alloys Compd..

[8] Kang T.W., Choi Y.B., Kang C.H., Park Y.J., Kim J.H., Bae B., Kim S.W. (2023). Development of NaY_9_Si_6_O_26_:Yb^3+^ Phosphors with High Thermal Stability for NIR Anti-Counterfeiting: Study of Its Crystal Structure and Luminescent Properties. RSC Adv..

[9] Zheng L., Zheng B., Xia H., Wang J., Song H., Chen B. (2021). Color-Tunable Emission and Non-Contact Optical Temperature Sensing Performance in NaY_9_Si_6_O_26_: Ce^3+^, Eu^3+^ Phosphors. Mater. Res. Bull..

[10] Zheng L., Zhou X., Zhang J., Xia H., Song H., Chen B. (2020). Multiple Occupation Sites of Bi^3+^ and Full Color Luminescence Tuning through Co-Doped Eu^3+^ in NaY_9_Si_6_O_26_ Phosphors. J. Alloys Compd..

[11] Brgoch J., Borg C.K.H., Denault K.A., Douglas J.R., Amanda Strom T., DenBaars S.P., Seshadri R. (2013). Rapid Microwave Preparation of Cerium-Substituted Sodium Yttrium Silicate Phosphors for Solid State White Lighting. Solid State Sci..

[12] Preparation Method of Fusiform Apatite Phase Y_4.67_(SiO_4_)_3_O Powder, CN105776231A. https://patents.google.com/patent/CN105776231A/en?oq=CN105776231A.

[13] Wang Q.-G., Huang J.-F., Zhou L., Wu W.-C., Cao L.-Y. (2018). Thermal Property of Y_4.67_ (SiO_4_)_3_ Ceramic Sintered from Hydrothermally Synthesized Spindle-like Y_4.67_(SiO_4_)_3_O Apatite Crystallites. J. Inorg. Mater..

[14] Gu H., Hou X., Zhang R., Fang D. (2019). Novel High-temperature-resistant Y_2_SiO_5_ Aerogel with Ultralow Thermal Conductivity. Int. J. Appl. Ceram. Technol..

[15] Boyer D., Derby B. (2003). Yttrium Silicate Powders Produced by the Sol–Gel Method, Structural and Thermal Characterization. J. Am. Ceram. Soc..

[16] Krsmanović R.W., Andrić Ž., Marinović-Cincović M., Zeković I., Dramićanin M.D. (2007). Optical and Thermal Investigation of Sol-Gel Derived Eu^3+^: Y_2_SiO_5_ Nanoparticles. Acta Phys. Polonica. A.

[17] Moya J.S., Díaz M., Serna C.J., Mello-Castanho S. (1998). Formation of Nanocrystalline Yttrium Disilicate Powder by an Oxalate Gel Method. J. Eur. Ceram. Soc..

[18] Jo S.H., Muralidharan P., Kim D.K. (2009). Low-Temperature Sintering of Dense Lanthanum Silicate Electrolytes with Apatite-Type Structure Using an Organic Precipitant Synthesized Nanopowder. J. Mater. Res..

[19] Tao S., Irvine J.T.S. (2001). Preparation and Characterisation of Apatite-Type Lanthanum Silicates by a Sol-Gel Process. Mater. Res. Bull..

[20] Li J., Cai Q., Horri B.A. (2023). Synthesis and Densification of Mo/Mg Co-Doped Apatite-type Lanthanum Silicate Electrolytes with Enhanced Ionic Conductivity. Chem. A Eur. J..

[21] Shannon R.D. (1976). Revised Effective Ionic Radii and Systematic Studies of Interatomic Distances in Halides and Chalcogenides. Acta Crystallogr. A.

[22] Lee F.C., Marr J., Glasser F.P. (1981). Compounds in the Na_2_O Y_2_O_3_ SiO_2_ System. Ceram. Int..

[23] Redhammer G.R., Roth G. (2003). Lithium and Sodium Yttrium Orthosilicate Oxyapatite, LiY_9_(SiO_4_)_6_O_2_ and NaY_9_(SiO_4_)_6_O_2_, at Both 100 K and near Room Temperature. Acta Crystallogr. C.

[24] Clark S.J., Segall M.D., Pickard C.J., Hasnip P.J., Probert M.I.J., Refson K., Payne M.C. (2005). First Principles Methods Using CASTEP. Z. Für Krist. -Cryst. Mater..

[25] Perdew J.P., Burke K., Ernzerhof M. (1996). Generalized Gradient Approximation Made Simple. Phys. Rev. Lett..

[26] Ceperley D.M., Alder B.J. (1980). Ground State of the Electron Gas by a Stochastic Method. Phys. Rev. Lett..

[27] Binnemans K., Görller-Walrand C. (1996). Application of the Eu^3+^ ion for site symmetry determination. J. Rare Earths.

[28] Malta O.L., Antic-Fidancev E., Lemaitre-Blaise M., Milicic-Tang A., Taibi M. (1995). The Crystal Field Strength Parameter and the Maximum Splitting of the ^7^F_1_ Manifold of the Eu^3+^ Ion in Oxides. J. Alloys Compd..

[29] Monteil A., El-Jouad M., Alombert-Goget G., Chaussedent S., Gaumer N., Mahot A., Chiasera A., Jestin Y., Ferrari M. (2008). Relationship between Structure and Optical Properties in Rare Earth-Doped Hafnium and Silicon Oxides: Modeling and Spectroscopic Measurements. J. Non-Cryst. Solids.

[30] Zhu C., Monteil A., EI-Jouad M., Gaumer N., Chaussedent S. (2010). Influence of Thermal Treatment on Optical and Structure Properties of Europium-Doped SiO_2_–HfO_2_ Glasses. J. Am. Ceram. Soc..

[31] Ćirić A., Stojadinović S., Sekulić M., Dramićanin M.D. (2019). JOES: An Application Software for Judd-Ofelt Analysis from Eu^3+^ Emission Spectra. J. Lumin..

[32] Ćirić A., Marciniak Ł., Dramićanin M.D. (2022). Self-Referenced Method for the Judd–Ofelt Parametrisation of the Eu^3+^ Excitation Spectrum. Sci. Rep..

[33] Binnemans K. (2015). Interpretation of Europium(III) Spectra. Coord. Chem. Rev..

[34] Walsh B.M., Di Bartolo B., Forte O. (2006). Judd-Ofelt Theory: Principles and Practices. Advances in Spectroscopy for Lasers and Sensing.

